# Risk Factors for SARS-CoV-2 Infection, Pneumonia, Intubation, and Death in Northeast Mexico

**DOI:** 10.3389/fpubh.2021.645739

**Published:** 2021-07-05

**Authors:** Hid Felizardo Cordero-Franco, Laura Hermila De La Garza-Salinas, Salvador Gomez-Garcia, Jorge E. Moreno-Cuevas, Javier Vargas-Villarreal, Francisco González-Salazar

**Affiliations:** ^1^Unidad de Investigación Epidemiológica y en Servicios de Salud, Delegación Nuevo León, Instituto Mexicano del Seguro Social, Monterrey, Mexico; ^2^Centro de Investigaciones Biomédicas Del Noreste, Instituto Mexicano Del Seguro Social, Monterrey, Mexico; ^3^Planeación y Enlace Institucional, Delegación Regional Nuevo León, Instituto Mexicano del Seguro Social, Monterrey, Mexico; ^4^Coordinación de Información y Análisis estratégico, Delegación Regional Nuevo León, Instituto Mexicano del Seguro Social, Monterrey, Mexico; ^5^División de Ciencias de La Salud, Departamento de Ciencias Básicas, Universidad de Monterrey, Monterrey, Mexico

**Keywords:** COVID-19, SARS-CoV-2, coronavirus, pneumonia, intubation endotracheal, death, Mexico

## Abstract

Despite the social distancing and mobility restriction measures implemented for susceptible people around the world, infections and deaths due to COVID-19 continued to increase, even more so in the first months of 2021 in Mexico. Thus, it is necessary to find risk groups that can benefit from more aggressive preventive measures in a high-density population. This is a case-control study of suspected COVID-19 patients from Nuevo León, Mexico. Cases were: (1) COVID-19-positive patients and COVID-19-positive patients who (2) developed pneumonia, (3) were intubated and (4) died. Controls were: (1) COVID-19-negative patients, (2) COVID-19-positive patients without pneumonia, (3) non-intubated COVID-19-positive patients and (4) surviving COVID-19-positive patients. ≥ 18 years of age, not pregnant, were included. The pre-existing conditions analysed as risk factors were age (years), sex (male), diabetes mellitus, hypertension, chronic obstructive pulmonary disease, asthma, immunosuppression, obesity, cardiovascular disease, chronic kidney disease and smoking. The Mann-Whitney *U* tests, Chi square and binary logistic regression were used. A total of 56,715 suspected patients were analysed in Nuevo León, México, with 62.6% being positive for COVID-19 and, of those infected, 14% developed pneumonia, 2.9% were intubated and 8.1% died. The mean age of those infected was 44.7 years, while of those complicated it was around 60 years. Older age, male sex, diabetes, hypertension, and obesity were risk factors for infection, complications, and death from COVID-19. This study highlights the importance of timely recognition of the population exposed to pre-existing conditions to prioritise preventive measures against the virus.

## Introduction

Currently, coronavirus disease 2019 (COVID-19) is a global public health problem. As of May 10, 2021, 158,551,526 people have been infected and 3,296,855 have died from this disease worldwide (1). The situation is no less dire in Mexico, where 2,366,496 infected people and 219,089 deaths have been documented (2). Since the World Health Organization (WHO) first declared COVID-19 a pandemic (3), and given the absence of a vaccine and effective treatment at that time, several studies have been conducted to determine the factors associated with severity and mortality. These studies demonstrated that older age and certain chronic conditions such as diabetes, hypertension and obesity, among others, were associated with higher risks of complications and death (4–9). Given the above findings, many countries, including Mexico, implemented preventive strategies aimed at reducing the exposure of vulnerable people, such as social distancing of the elderly and people with chronic diseases, closure of non-essential companies and schools, limits on the number of people in stores and essential establishments, educational reinforcement of handwashing and the use of face masks. These measures aimed to reduce the rate of infections and subsequently the mortality associated with the causal virus, however, they had significant growth in January and February of 2021 (1). Furthermore, a recently published North American study was unable to identify reductions in mortality during restricted mobility days in either the United States or Europe (10). The lack of control of the rates of infection and death is a clear example that there is much we still do not understand about the transmissibility of COVID-19 since many people around the world continue becoming infected and infecting others.

Nuevo León is a state of Mexico with a population density greater than the national average (11). It is a highly industrialised region that includes approximately 2.54 million economically active people (12). The closure of many productive activities due to the pandemic caused the loss of thousands of jobs, which triggered a growth in informal economic activities (13). In many cases, these circumstances prevented home office work, which could cause greater exposure to infected people and a greater spread of COVID-19 in this population (14). Moreover, this state has a high prevalence of chronic diseases such as obesity, diabetes and hypertension (15), which have consistently been associated with infection (16, 17) and worse outcomes in the course of this disease (4–9, 18, 19).

Therefore, it is imperative to determine not only the factors associated with a worse outcome of this disease but also those related to infection. This knowledge is necessary in a high-density population to establish risk groups that could benefit from more aggressive preventive measures, including vaccines.

The aim of this work was to determine the risk factors associated with COVID-19 infection and to determine the risk factors for pneumonia, intubation and death among those infected with COVID-19.

## Materials and Methods

### Study Population

The data from patients in Nuevo León, Mexico, with suspected COVID-19 were obtained from the open access database of the General Directorate of Epidemiology of the Ministry of Health. The database contains data from all health institutions in Mexico. For this study, data collected from February 11, 2020 to September 24, 2020 were analysed (20).

### Groups and Study Goals

Cases were defined as follows: (1) COVID-19-positive patients and COVID-19-positive patients who (2) developed pneumonia, (3) were intubated and (4) died. The respective controls were (1) COVID-19-negative patients, (2) COVID-19-positive patients without pneumonia, (3) non-intubated COVID-19-positive patients and (4) surviving COVID-19-positive patients. The sample size for each study objective was sufficient to identify odds ratios of 1.14–1.47 with a confidence level of 95% and a statistical power of 100% (see Supplementary Figure 1 for a more detailed description of the size of each study group).

### Procedures

Patients aged ≥18 years who were treated in Nuevo León, Mexico, were included; pregnant patients were excluded (*n* = 513). Pre-existing conditions analysed included those previously identified as risk factors for COVID-19 and its complications: age (years), sex (male), diabetes mellitus, hypertension, chronic obstructive pulmonary disease, asthma, immunosuppression, obesity, cardiovascular disease, chronic kidney disease and smoking (4–9, 18, 19). The following outcomes were evaluated: COVID-19 infection [severe acute respiratory syndrome coronavirus 2 (SARS-CoV-2) polymerase chain reaction-positive], COVID-19 pneumonia, COVID-19 intubation and COVID-19 death. Except for age, all variables were coded in a categorical binary method (Yes vs. No). The following data were also analysed: medical care institution (public or private), origin from the metropolitan area and hospitalisation.

### Ethical Considerations

This protocol was subject to the institutional, national and international norms and regulations on research and ethics in health and was approved by the Local Committee on Ethics and Health Research of the Mexican Institute of Social Security No. 1909 and registered as R-2020-1909-027. In this study, an open access database available on the internet was managed, and non-personal information of the study patients was obtained or recorded.

### Statistical Analysis

The measures of central tendency and proportions, as well as 95% confidence intervals, were estimated. The Mann–Whitney *U* test was used to compare age between independent groups after the Kolmogorov–Smirnov test verified the non-normal distribution of variables. Univariate and multivariate odds ratios were estimated using Chi-square tests and binary logistic regressions, respectively. A *p*-value < 0.05 was considered significant.

## Results

From the beginning of the pandemic until September 24, 2020, 56,715 patients with suspected COVID-19 were registered in Nuevo León, of whom 85.1% lived in the metropolitan area. The most frequent comorbidity was hypertension, followed by obesity. Of the total population analysed, 62.6% were positive for COVID-19, and of these, 14% developed pneumonia, 20.7% were hospitalised, 2.9% were intubated and 8.1% died (Table 1).

**Table 1:** General data, comorbidities and outcomes of patients analysed for SARS-CoV-2 in Nuevo León (February to September 2020).

	***N* = 56,715**	**%**
Live in metropolitan area	48,274	85.1%
**Gender**		
Female	27,608	48.7%
Male	29,107	51.3%
**Institution**		
Public	53,709	94.7%
Private	3,006	5.3%
**Comorbidities**		
Hypertension	9,489	16.7%
Obesity	8,504	15.0%
Diabetes mellitus	7,874	13.9%
Smoking	4,627	8.2%
Asthma	1,536	2.7%
Another comorbidity	1,405	2.5%
Cardiovascular disease	1,165	2.1%
Chronic kidney disease	897	1.6%
Immunosuppression	700	1.2%
COPD	491	0.9%
COVID-19 positive patients	35,476	62.6%
Pneumonia	4,974	14.0%^*^
Hospitalised	7,351	20.7%^*^
Intubated	1,021	2.9%^*^
Dead	2,860	8.1%^*^

*COPD, chronic obstructive pulmonary disease*.

* *Proportions of all COVID-19 positive patients*.

The mean age of the infected patients was 44.7 years, whereas the mean ages of those who developed pneumonia, were intubated, or died were 57.5, 60.1, and 63.6 years, respectively. In all comparisons, the cases were older than the controls (Table 2).

**Table 2:** Comparison of patient age related to the main output variables.

	**Mean age (years)**	**CI 95%**	***P*-value**
COVID-19 positive	44.7	44.6–44.9	<0.0001
COVID-19 negative	42.2	42.0–42.4	
Pneumonia	57.5	57.1–57.9	<0.0001
No Pneumonia	42.1	41.9–42.2	
Intubated	60.1	59.3–60.9	<0.0001
No Intubated	57.2	56.8–57.5	
Dead	63.6	63.1–64.0	<0.0001
Survivors	42.6	42.4–42.7	

*CI, confidence interval*.

At the univariate level, male sex, diabetes, hypertension, and obesity were shown to be significantly associated with the risk of COVID-19 infection, pneumonia, intubation and death. Conversely, chronic obstructive pulmonary disease, immunosuppression, cardiovascular disease and chronic kidney disease showed a protective effect against COVID-19 infection, but were risk factors for pneumonia and death, while smoking was a protective factor against infection and those complications. Asthma showed a protective effect against infection but did not show any association with complications (Table 3).

**Table 3:** Univariate analysis of pre-existing conditions associated with confirmed COVID-19 infection, pneumonia, intubation, and death.

**Output variable**	**COVID-19 positive**	**Pneumonia**	**Intubation**	**Death**
Pre-existing conditions	OR (CI 95%)	OR (CI 95%)	OR (CI 95%)	OR (CI 95%)
Male	1.17 (1.13–1.21)^**^	1.45 (1.36–1.54)^**^	1.14 (0.99–1.31)	1.47 (1.36–1.59)^**^
Diabetes mellitus	1.59 (1.51–1.68)^**^	3.75 (3.50–4.00)^**^	1.21 (1.05–1.38)^*^	4.17 (3.85–4.53)^**^
COPD	0.63 (0.53–0.75)^**^	4.09 (3.18–5.28)^**^	1.01 (0.64–1.60)	5.74 (4.40–7.48)^**^
Asthma	0.64 (0.58–0.71)^**^	1.02 (0.83–1.24)	1.10 (0.71–1.70)	0.97 (0.75–1.26)
Immunosuppression	0.51 (0.44–0.60)^**^	2.26 (1.77–2.89)^**^	1.41 (0.93–2.13)	2.89 (2.20–3.80)^**^
Hypertension	1.40 (1.34–1.47)^**^	3.68 (3.44–3.92)^**^	1.40 (1.22–1.60)^**^	4.85 (4.48–5.25)^**^
Cardiovascular disease	0.74 (0.66–0.83)^**^	3.48 (2.95–4.10)^**^	1.13 (0.83–1.53)	4.16 (3.47–4.98)^**^
Obesity	1.69 (1.61–1.78)^**^	1.71 (1.60–1.84)^**^	1.35 (1.17–1.56)^**^	1.74 (1.59–1.90)^**^
Chronic kidney disease	0.83 (0.72–0.94)^*^	5.49 (4.61–6.54)^**^	1.30 (0.97–1.75)	7.43 (6.20–8.91)^**^
Smoking	0.78 (0.73–0.83)^**^	0.81 (0.72–0.92)^*^	1.17 (0.90–1.52)	0.82 (0.70–0.96)^*^

*OR, odds ratio; CI, confidence interval; COPD, chronic obstructive pulmonary disease*.

* *p < 0.05*;

** *p < 0.0001*.

The multivariate level showed similar trends to those of the univariate analysis: male sex, diabetes mellitus, hypertension and obesity were risk factors for COVID-19 infection, whereas older age also showed an association, but this association was only marginal (odds ratio, 1.01; 95% confidence interval, 1.00–1.02). These factors also showed a consistent risk association for complications, that is, pneumonia, intubation (except for diabetes) and death. Conversely, factors that were protective against COVID-19 infection did not show an association with complications (such as chronic obstructive pulmonary disease, asthma, and cardiovascular disease) or were risk factors for pneumonia and death (immunosuppression and chronic kidney disease); smoking was also protective against death (Table 4).

**Table 4:** Multivariate analysis of pre-existing conditions associated with confirmed COVID-19 infection, pneumonia, intubation, and death.

**Outcome variable**	**COVID-19 positive**	**Pneumonia**	**Intubated**	**Death**
Pre-existing conditions	OR (CI 95%)	OR (CI 95%)	OR (CI 95%)	OR (CI 95%)
Age (risk for each year added)	1.01 (1.00–1.02)^**^	1.06 (1.05–1.07)^**^	1.01 (1.00–1.02)^**^	1.08 (1.07–1.09)^**^
Male	1.21 (1.17–1.25)^**^	1.65 (1.54–1.76)^**^	1.23 (1.07–1.41)^*^	1.74 (1.59–1.89)^**^
Diabetes mellitus	1.38 (1.30–1.46)^**^	1.65 (1.53–1.79)^**^	1.04 (0.89–1.20)	1.52 (1.38–1.68)^**^
COPD	0.53 (0.44–0.63)^**^	1.13 (0.85–1.49)	0.83 (0.51–1.33)	1.29 (0.95–1.74)
Asthma	0.66 (0.59–0.73)^**^	1.18 (0.95–1.47)	1.10 (0.70–1.72)	1.17 (0.88–1.57)
Immunosuppression	0.48 (0.41–0.55)^**^	1.48 (1.13–1.94)^*^	1.46 (0.96–2.22)	1.92 (1.41–2.61)^**^
Hypertension	1.11 (1.05–1.17)^**^	1.27 (1.17–1.38)^**^	1.20 (1.03–1.39)^*^	1.43 (1.30–1.57)^**^
Cardiovascular disease	0.59 (0.52–0.67)^**^	1.11 (0.92–1.33)	0.90 (0.66–1.24)	1.02 (0.83–1.26)
Obesity	1.67 (1.58–1.76)^**^	1.56 (1.44–1.70)^**^	1.40 (1.20–1.63)^**^	1.63 (1.48–1.81)^**^
Chronic kidney disease	0.65 (0.57–0.75)^**^	2.23 (1.83–2.72)^**^	1.14 (0.84–1.55)	2.86 (2.32–3.53)^**^
Smoke	0.76 (0.71–0.81)^**^	0.81 (0.71–0.93)^*^	1.08 (0.82–1.42)	0.81 (0.68–0.97)^*^

*OR, odds ratio; CI, confidence interval; COPD, chronic obstructive pulmonary disease*.

* *p < 0.05*;

** *p < 0.0001*.

## Discussion

This study, which is based on a large representative database, examines pre-existing factors associated with COVID-19 infection as well as pneumonia, intubation, and death in northern Mexico. The main risk factors for infection and its complications were older age, male sex, hypertension, and obesity. Thus, it is possible to stratify patients exposed to these conditions to reinforce preventive and therapeutic strategies.

The population analysed in this study primarily corresponds to the metropolitan area of Nuevo León, a region with a high population density (11), which is a determining factor in the risk of contagion (21). Compared with another study performed in the total Mexican population, Nuevo León registered a higher proportion of positive cases of COVID-19 (62.6 vs. 52.6%) (6). However, the rate of hospitalisations in this state was lower than that reported in the previous study (35.3%) (6), which may be due to the fact that many outpatient diagnostic tests were performed in this state.

An important finding of this study is the mean age of the infected patients, which was younger than that of those who developed complications. This agrees with the estimates of Xiong et al. (14) in California, who found that the highest infection rate was in the population aged 18–59 years, that is, the economically active population, but there was a higher fatality rate in the population aged ≥60 years. In accordance with this finding, the higher infection rate in younger individuals seems to be related to the need to work outside home (14), whereas the higher severity and lethality seems to be explained by immunosenescence, a chronic low-grade systemic inflammation that has been proposed as a common factor resulting in greater susceptibility to COVID-19 severity (22).

Another factor consistently associated with infection and severity was male sex. In this regard, men have been described as having biological and behavioural differences that place them at a higher risk than women, such as their higher level of testosterone that inhibits antibody production, the higher presence of receptors of angiotensin-converting enzyme 2 that facilitate viral anchorage and replication, insufficient handwashing practices, non-adherence to health services and reluctance to follow public health measures (17, 23–25).

In this population, pre-existing conditions did not equally affect the risks of infection, pneumonia, intubation, and death. Notably, chronic obstructive pulmonary disease, asthma, cardiovascular disease and even smoking showed protective effects against infection but did not show any association with complications or, as in the case of smoking, this was a “protective” factor against death. More remarkable still, factors such as immunosuppression and chronic kidney disease, which acted as protectors against infection, increased the risk of pneumonia and death. This contradiction could be explained because, as mentioned before, the Mexican health authorities implemented quarantine measures for the vulnerable population, which most likely made these groups under-represented in this database. In other words, it is not necessarily true that having such comorbidities offers any protection against COVID-19 but rather that the susceptible population was less exposed to the virus. In fact, diabetes, immunosuppression and chronic kidney disease, three serious medical conditions, have been reported to increase the risk of severity and death from COVID-19 (6, 9, 26). Therefore, preventive measures should be continued and reinforced in patients with these comorbidities.

An increased risk of infection became apparent among people with diabetes, hypertension, and obesity. Additionally, these comorbidities were risk factors for pneumonia, intubation (not diabetes) and death. This triad of comorbidities has already been described as a risk factor for adverse outcomes from COVID-19 (6, 9, 27). At this point, it is necessary to emphasise the role of these chronic diseases in susceptibility to contagion. Yadav et al. had already pointed out this relationship in the Mumbai population and noted that the concurrence of these chronic diseases was strongly associated with COVID-19 infection (16). The biological explanation is that these comorbidities can lead to the overexpression of SARS-CoV-2 receptor molecules, such as angiotensin-converting enzyme 2 and CD147 (17). The practical implication of this relationship is that in a high-density population, with a high prevalence of these comorbidities, the risk of spreading the virus, even with social distancing measures, is extremely high. These findings emphasise the need to prioritise preventive measures such as vaccination in the potentially more vulnerable groups.

A limitation of this study is its use of a secondary data source. Thus, it is possible that some data were under-represented; therefore, the results shown should be viewed with caution. However, a strength of this study is that it included almost 100% of the data in a highly industrialised area with a high population density.

In conclusion, older age, male sex, hypertension, and obesity were factors consistently associated with COVID-19 infection as well as its complications, including pneumonia, intubation and death. Additionally, diabetes mellitus, immunosuppression and chronic kidney disease were identified as risk factors for pneumonia and death from COVID-19. This study highlights the importance of timely recognition of the population exposed to pre-existing conditions to prioritise preventive measures against the virus, such as mass vaccination.

## Data Availability Statement

The datasets presented in this study can be found in online repositories. The names of the repository/repositories and accession number(s) can be found at: COVID-19 open access database of the General Directorate of Epidemiology of the Ministry of Health.

## Ethics Statement

The studies involving human participants were reviewed and approved by 1909-Local Committee of ethics on research, Mexican Institute of Social Security, Monterrey, Nuevo Leon, Mexico. Written informed consent for participation was not required for this study in accordance with the national legislation and the institutional requirements.

## Author Contributions

HC-F: data analysis, elaboration, and review of manuscript. FG-S: wrote protocol, wrote manuscript manage data, traduction, edition, and review final version. JV-V: design protocol, registry, and review final version. JM-C: design protocol, discussion, and review manuscript final. SG-G: get data, manage variables, and review final version manuscript. LD: registry protocol, get data, and review of final manuscript. All authors contributed to the article and approved the submitted version.

## Conflict of Interest

The authors declare that the research was conducted in the absence of any commercial or financial relationships that could be construed as a potential conflict of interest.
