# Supplementary material for: Risk Factors for SARS-CoV-2 Infection, Pneumonia, Intubation, and Death in Northeast Mexico
Source: Front Public Health. 2021 Jul 5;9:645739. doi: 10.3389/fpubh.2021.645739 (PMC8287121; doi:10.3389/fpubh.2021.645739)
Supplement: Supplementary file 1 [file Data_Sheet_1.pdf]

Supplementary Material

1.1 Supplementary Figures

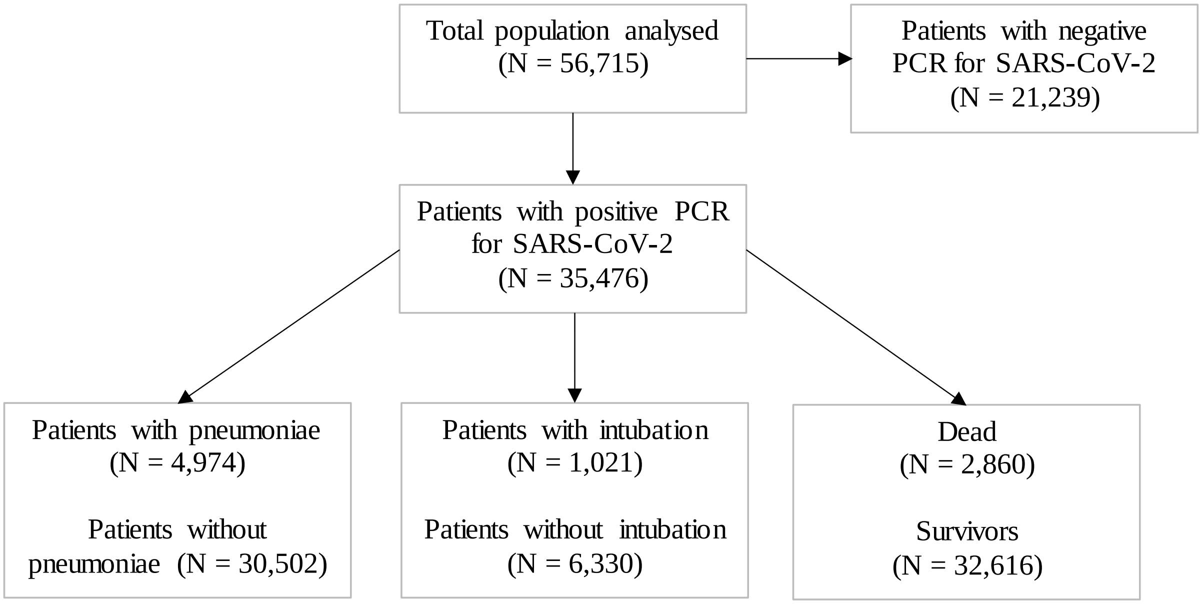

Supplementary Figure 1. Flow diagram of the study population. (PCR: polymerase chain reaction).
